# Stroke risk in rheumatoid arthritis patients: exploring connections and implications for patient care

**DOI:** 10.1007/s10238-023-01288-7

**Published:** 2024-01-31

**Authors:** Ola A. Al-Ewaidat, Moawiah M. Naffaa

**Affiliations:** 1 Department of Internal Medicine, Ascension Saint Francis Hospital, Evanston, IL 60202 USA; 2 Department of Psychology and Neuroscience, Duke University, Durham, NC 27708 USA; 3 Department of Cell Biology, Duke University School of Medicine, Durham, NC 27710 USA

**Keywords:** Stroke, Rheumatoid arthritis (RA), Chronic inflammation, Cardiovascular disease (CVD), Multi-biomarker disease activity (MBDA), Anti-cyclic citrullinated peptide (Anti-CCP2) test, Rheumatoid factor (RF)

## Abstract

Rheumatoid arthritis (RA) can independently increase the risk of stroke, affecting both young and adult RA patients. Recent attention has been drawn to the association between stroke and RA, supported by mounting evidence. Given that stroke is a significant and an urgent public health concern, this review aims to highlight the relationship between stroke and RA, covering mechanisms, underlying risk factors, early detection tools, and treatment implications. By uncovering the connection that links RA to stroke, we can pave the way for targeted healthcare practices and the development of preventive strategies for individuals with RA. Therefore, further research is imperative to deepen our understanding of this association and, ideally, guide treatment decisions for individuals at risk of both RA and stroke.

## Introduction

Stroke is a sudden loss of focal neurological function, comprising ischemic stroke (IS) and hemorrhagic stroke (HS) [1], and is a major public health concern. In 2019, it was the second leading cause of death globally, resulting in significant disability-adjusted life years lost [2]. The physical and emotional consequences for patients and their families are profound, underscoring the importance of understanding stroke risk [3]. Rheumatoid arthritis (RA), an autoimmune disease primarily affecting joints [4], is considered a significant factor contributing to an increased risk of stroke due to specific risk factors and underlying mechanisms [5]. There is an urgent need for a comprehensive understanding of this connection to help prevent RA patients from experiencing a heightened risk of stroke and its associated consequences.

It has been reported that patients with rheumatoid arthritis (RA) face an elevated risk of cardiovascular diseases, including myocardial infarction and stroke [6–9]. However, prior research has yielded inconsistent findings concerning the direct association between RA and the risk of stroke [10–13]. This connection between RA and stroke can be attributed to multiple factors, including underlying inflammatory processes and cardiac manifestations [14]. Cardioembolic strokes are prevalent among these patients, with their contribution to increased risk independently reported from traditional risk factors [14]. Furthermore, non-atherosclerotic vasculopathy has been identified as a significant contributor to ischemic stroke in RA patients [14, 15]. Consequently, comprehending the root causes of stroke in RA patients is essential for diagnosis and prevention, as well as for managing secondary stroke prevention. Achieving this goal involves the identification of diagnostic markers and tools for early detection and the utilization of disease-modifying antirheumatic drugs (DMARDs) in conjunction with standard therapy when necessary [16].

In this review, our primary aim is to elucidate the existing comprehension of the connection between stroke and RA, covering the underlying mechanisms, risk factors, diagnostic methods, and their implications for personalized treatment strategies. By attaining a profound insight into this correlation, we can optimize personalized treatment planning and strengthen stroke prevention measures for individuals living with RA. Moreover, by being aware of our current understanding of the link between RA and stroke, we can gain better clarity on the additional research needed to improve our comprehension of managing stroke risk in RA patients.

## The interconnected pathophysiology and mechanisms of stroke in RA

The precise mechanism of stroke in RA remains a subject of ongoing research and is not yet fully elucidated (Table 1). RA is a chronic inflammatory disease primarily characterized by inflammation within the synovial tissue in joints [17, 18]. This inflammation triggers the release of enzymes, pro-inflammatory factors, and cytokines, leading to the degradation of the synovial tissue [19–22]. The elevated levels of inflammatory cytokines in arthritis may play a critical role in linking arthritis and stroke. These cytokines can enter the bloodstream and increase the production of adhesion molecules and other pro-inflammatory molecules [23]. As a result, monocytes and leukocytes adhere to the endothelial cells of blood vessels, migrate into the vessel walls, and contribute to the development of atherosclerosis, which can eventually lead to stroke [24, 25]. Furthermore, the increased risk of stroke in individuals with rheumatoid arthritis is thought to be connected to vasculitis, a medical condition marked by inflammation of blood vessels, specifically impacting the medium and small cerebral vessels in the brain [26].

**Table 1:** Summarizing the potential mechanisms underlying stroke occurrence in patients with RA

References	RA’s underlying pathology can cause strokes	Descriptions
Wick et al. [22], Gonzalez-Gay et al. [19, 21], van Leuven et al. [20], Miller et al. [23]	Inflammatory cytokines—atherosclerosis	RA’s chronic inflammation induces cytokine release, leading to atherosclerosis and potentially initiating stroke
Rawla [26]	Vasculitis	When Vasculitis is linked to RA, it may contribute to an increased risk of stroke
Lindhardsen et al. [27]	Cardiac valve injury	RA can increase the risk of stroke by causing heart valve damage
Ross [28]	Immune-driven atherosclerosis	RA leads to stroke acceleration by promoting immune-mediated inflammatory processes that trigger atherosclerosis
Lima et al. [32], Vaudo et al. [33], Hallenbeck et al. [31], Akhmedov et al. [34]	Endothelial dysfunction	Endothelial dysfunction linked to RA: Early atherosclerosis pathway may elevate stroke risk
Lima et al. [32]	Conventional cardiovascular risk factors	RA patients frequently exhibit cardiovascular risk factors, raising stroke risk
Suss et al. [35]	Neuroinflammation	Neuroinflammation caused by RA is a potential factor in stroke occurrence
Tam et al. [36]	Elevated arterial stiffness	RA is associated with heightened arterial stiffness, further increasing stroke risk
Nishioku et al. [37]	Blood–brain barrier disruption	RA may disrupt the blood–brain barrier, potentially raising stroke risk

In addition, RA has other implications for cardiovascular health. RA can cause damage to heart valves and increase the risk of atrial fibrillation (Afib), a condition that significantly elevates the likelihood of stroke due to blood clot formation [27]. Furthermore, RA is associated with accelerated atherosclerosis, an immune-mediated inflammatory process [28]. The systemic inflammation present in autoimmune diseases like RA [29, 30] contributes to the progression of atherosclerosis. Endothelial dysfunction, which is an early pathological process in atherosclerosis, plays a significant role in the increased cardiovascular risk observed in systemic immune diseases, particularly RA. This dysfunction is often associated with elevated levels of TNF-alpha. As a result, anti-TNF-alpha treatments are considered to reduce articular symptoms and decrease cardiovascular risk [31–34].

Additionally, patients with autoimmune diseases such as RA are more likely to exhibit traditional cardiovascular risk factors [32], which further compounds the overall cardiovascular risk in individuals with RA. Moreover, RA has been extensively linked to the pathophysiology of stroke, involving factors such as neuroinflammation [35], increased arterial stiffness [36], and disruption of the blood–brain barrier [37]. These factors contribute to the overall risk and impact of stroke in individuals with RA.

While the association between RA and stroke is established, there is still much to learn about the underlying mechanisms. Further research is needed to gain a deeper understanding of these complex interactions and to develop targeted strategies for the early treatment and prevention of ischemic stroke in patients with RA. By identifying and addressing the specific mechanisms involved, it may be possible to develop more effective interventions to reduce the risk of stroke in individuals with RA. This could include optimizing the management of RA-related inflammation, controlling cardiovascular risk factors, and exploring targeted therapies that modulate the immune response or inhibit specific cytokines involved in the pathogenesis of both RA and ischemic stroke.

Furthermore, studies have demonstrated that inflammatory conditions other than RA, such as ankylosing spondylitis, are also linked to an increased risk of stroke [38, 39]. Therefore, future investigations are crucial in identifying whether neuroinflammatory agents, such as TNF-a, IL-6, or IL-1b, contribute to the association of stroke risk with RA and whether these agents are similarly influential in other inflammatory diseases, such as ankylosing spondylitis. Understanding these connections will aid in comprehending the factors contributing to stroke risk in various inflammatory diseases and in establishing common or distinct preventive and management strategies for different inflammatory conditions.

## The link between stroke and RA: analysis of risk factors and associations

In recent years, there has been a significant amount of research conducted on stroke risk factors. Initially, researchers primarily focused on traditional factors such as hypertension, diabetes, obesity, smoking, alcoholism, and physical inactivity. However, recent studies have uncovered additional risk factors, providing valuable insights [40, 41]. Researchers have conducted quantitative meta-analyses, which have identified several stroke risk factors. These include migraine, anemia, inflammatory bowel disease, sleep insufficiency, inadequate fruit and vegetable intake, and inflammatory diseases [42–46].

Additionally, extensive research has investigated the association between stroke pathogenesis and RA, revealing a connection rooted in shared inflammatory and immune mediators that affect brain blood vessels [47]. Moreover, RA patients have a 1.5-fold increased risk of cardiovascular disease, emphasizing the elevated risk of stroke [48, 49]. Cardiac complications like coronary vasculitis, arrhythmias, and valvular heart diseases contribute to cardioembolic stroke in individuals with RA [14]. Importantly, this increased stroke risk remains independent of traditional risk factors, including age, sex, smoking, alcohol consumption, hypertension, diabetes, obesity, sleep apnea, sedentary lifestyle, and dyslipidemia [14].

Specifically, a meta-analysis investigating stroke risk in rheumatic inflammatory diseases, with a particular emphasis on RA, has revealed a significant association between RA and an increased risk of stroke [50]. Another systematic review and meta-analysis, accounting for factors such as age, sex, and traditional risk factors, demonstrated that individuals with arthritis have a 36% higher risk of stroke compared to the general population [38]. Further subgroup analysis within this study indicated a 53% higher risk of ischemic stroke and a 45% higher risk of hemorrhagic stroke among arthritis patients. Notably, the highest risk of stroke was observed in younger individuals with arthritis (Table 2) [38].

**Table 2:** Summarizing studies show a connection between stroke and rheumatoid arthritis (RA) patients

References	Type of study	Findings involving risk factors, therapies, or biomarkers
Solomon et al. [139]	Population-based cohort study	Among individuals with rheumatoid arthritis, young adults, and those with no prior history of CVD events exhibit the highest rate ratio for CVD events, which probably includes a higher risk for stroke as well. Nevertheless, when considering absolute terms, the disparity in event rates is most pronounced in older adults
Nadareishvili et al. [5]	Nested case–control analysis	The severity of RA is associated with an increased risk of stroke, particularly ischemic stroke. In the context of RA treatment, Rofecoxib appears to be linked to a higher risk of stroke, while the impact of corticosteroids remains uncertain in this study
Solomon et al. [54]	Prospective longitudinal cohort study	The rate ratio for CVD events is highest among young adults with RA and those without a history of prior CVD events, which may potentially increase the likelihood of stroke occurrences. However, when looking at absolute terms, the disparity in event rates is greatest among older adults
Micha et al. [56]	Systematic review and meta-analysis	Treating inflammation with methotrexate in diseases like RA may reduce the risk of cardiovascular disease and the occurrence of strokes. In essence, addressing inflammation directly may lower the risk of strokes
Barnabe et al. [57]	Observational cohorts and randomized controlled trials (RCTs)	Therapies targeting anti-tumor necrosis factor α (anti-TNFα therapy) are linked to a decreased risk of all cardiovascular events, which may potentially reduce the risk of strokes in individuals diagnosed with rheumatoid arthritis
Avina-Zubieta et al. [52]	Comprehensive meta-analysis	Roughly 50% of patients with RA encountered an elevated risk of CVD, which is likely associated with an increased occurrence of strokes
Lindhardsen et al. [27]	Nationwide cohort study	Rheumatoid arthritis was linked to a higher incidence of atrial fibrillation and stroke. Relative risks increased across various age groups, indicating higher relative risks among younger patients, while older patients exhibited more significant absolute differences in risk
Wiseman et al. [50]	Systematic Review and Meta-Analysis	RA is significantly associated with an elevated risk of both ischemic and hemorrhagic strokes, especially in individuals aged below 50 years. This may indicate heightened RA activity occurring before effective inflammation control is achieved
Fransen et al. [51]	Meta-Analysis	The relative risk of stroke in rheumatoid arthritis (RA) patients does not exhibit significant gender-related differences. Among RA patients, those under the age of 50 have the highest relative risk, while older patients exhibit a relatively lower risk
Lee et al. [53]	cohort study	RA patients who are seropositive face a heightened risk of ischemic stroke. In hypertensive females without other traditional risk factors for stroke, there is an increased risk of stroke, which could potentially be linked to RA
Liu et al. [38]	A systematic review and meta-analysis of cohort studies	Individuals diagnosed with arthritis face an approximately 50% greater risk of experiencing both ischemic and hemorrhagic strokes when compared to their healthy counterparts. The risk of stroke did not differ significantly between sexes, and the risk was higher in individuals under 45 years of age compared to those above 65 years of age
Trommer et al. [39]	Nationwide cohort study	RA is associated with both stroke and transient ischemic attack (TIA), significantly heightening the risk for younger patients
Kang et al. [58]	Nationwide cohort study	The association between CVD and RA was found to be more pronounced in females and younger individuals, potentially posing a risk for stroke. Additionally, a significant association was found between ischemic stroke and RA in non-diabetic patients

Interestingly, previous meta-analyses have also suggested that younger arthritis patients face the highest risk of stroke, even in the presence of fewer traditional risk factors [51]. This elevated risk, particularly prominent in individuals aged 50 years or younger, contributes significantly to the occurrence of strokes in young RA individuals [50]. These findings emphasize that RA is an independent risk factor for stroke.

A comprehensive meta-analysis involving seven studies and a total of 39,520 patients diagnosed with rheumatoid arthritis found a 41% increase in the risk of stroke among patients with RA [52]. Furthermore, a Korean cohort study emphasized an increased association between seropositive RA and ischemic stroke [53]. The study stressed the importance of screening to improve outcomes in specific RA patient populations, such as females with hypertension, non-diabetes, and non-dyslipidemia [53]. Another prospective longitudinal cohort study highlighted that alongside traditional cardiovascular risk factors, RA severity markers are associated with higher cardiovascular risk in RA patients [54]. Conversely, reduced RA disease activity and the use of medications like methotrexate (MTX) and TNF-alpha inhibitors are associated with fewer cardiovascular events, including strokes (Table 2) [55–57].

A nested case–control analysis within a longitudinal databank of 16,990 patients with rheumatoid arthritis (RA) revealed that RA patients have a higher risk of ischemic stroke compared to individuals with noninflammatory rheumatic disease [5]. The study identified risk factors for ischemic stroke, including the severity of RA, hypertension, myocardial infarction, and low-dose aspirin use. However, diabetes, smoking, exercise, and body mass index did not show a significant association with the risk of ischemic stroke [5]. A population-based study of 25,385 Canadian adults with RA, compared to age- and sex-matched controls, revealed a significant absolute increase in stroke risk among patients with RA [9]. Similarly, a nationwide Danish cohort study involving 18,247 patients with RA found an increased incidence of stroke compared with the general population [27]. Although the extent to which atrial fibrillation contributed to stroke risk was not determined, the study also highlighted a higher risk of atrial fibrillation in patients with RA (Table 2) [27].

A recent study conducted in Germany has established a significant correlation between RA and both stroke and transient ischemic attack (TIA). The research also emphasized that young patients, in particular, are at a heightened risk of experiencing these conditions in connection with RA [39]. In another nationwide comprehensive study, a notable association between rheumatoid arthritis (RA) and an increased risk of stroke was discovered, even after meticulous adjustment for numerous potential confounding variables. Specifically, individuals with RA exhibited a 23% higher likelihood of experiencing a stroke compared to their counterparts without RA (Table 2) [58].

A systematic review and meta-analysis revealed a distinct trend of increased stroke risk in individuals younger than 50 years, implying that atherosclerosis may not be the exclusive underlying cause. It is possible that systemic inflammation plays a role, and the elevated risk in younger age groups could be indicative of heightened rheumatoid arthritis (RA) activity before inflammation is effectively managed [50].

The above studies provide growing evidence supporting an increased risk of stroke in individuals with RA. Both traditional cardiovascular risk factors and markers of RA severity contribute to this elevated risk. Ongoing research aims to further understand the connections between RA and stroke risk factors, which can contribute to the development of targeted healthcare practices for individuals with RA.

Furthermore, future research can encompass systematic reviews, meta-analyses, and epidemiological studies to explore the relationship between biomarkers, cytokines, autoantibodies, various inflammatory and neuroinflammatory agents, and the severity of RA concerning the diverse risks of stroke occurrence across different patient groups, encompassing age, sex, and other traditional factors. These urgently needed studies aim to investigate whether there are other key interacting markers or agents that warrant further investigation and research.

## Is there an association between anti-cyclic citrullinated protein test and stroke risk in relation to arthritis activity

Anti-citrullinated peptide antibodies (ACPA), primarily detected using the second-generation anti-cyclic citrullinated peptide test (anti-CCP2), are a distinctive hallmark in patients with RA [59]. Anti-cyclic citrullinated proteins (Anti-CCPs) play a pivotal role in the inflammatory and proatherogenic status observed in individuals with RA [60]. Strategically targeting these autoantibodies may offer a promising approach for preventing the development of cardiovascular diseases including stroke in RA patients [60].

The presence of elevated levels of anti-CCP antibodies is correlated with more severe clinical outcomes in rheumatoid arthritis (RA), increased disease activity, and a heightened risk of worse radiographic progression [61–64]. Retrospective studies have explored their predictive value, revealing that anti-CCP antibodies can be detected in the serum of individuals who subsequently develop RA up to fourteen years prior to the onset of clinical symptoms, with antibody titers progressively rising as disease onset approaches [65]. These findings have been replicated in studies involving patients with early-stage RA, thus reaffirming the clinical utility of anti-CCP antibodies as a diagnostic and prognostic tool for individuals experiencing RA symptoms lasting less than one or two years [59, 66].

The 2010 RA Classification Criteria have been subsequently updated to enable earlier RA diagnosis by incorporating the detection of anti-CCP antibodies as a pivotal criterion for diagnosing the disease [67]. The sensitivity values for detecting ACPA are among the highest achievable, largely due to the strong association between ACPA production and genetic factors [68–70]. Currently, the anti-CCP2 antibody test exhibits superior specificity and, in many cases, comparable or even superior sensitivity when compared to rheumatoid factor (RF) or other ACPAs [66].

Anti-CCP testing has been found to hold significant value in assessing the health risks of CVD, including stroke, in patients with RA. In a study involving postmenopausal women participating in the Women’s Health Initiative (WHI), researchers observed a heightened risk of coronary heart disease (CHD), stroke, and total CVD among individuals with RA. Furthermore, the risk of fatal CVD was notably elevated, particularly among those who tested positive for anti-CCP antibodies. Interestingly, this study has shown that the presence of anti-CCP antibodies or RF positivity did not demonstrate a significant correlation with CVD morbidity or mortality. However, the risks of both CHD and CVD were closely associated with traditional CVD risk factors and the presence of joint pain in these women with RA. Notably, inflammation in women with RA was strongly linked to an increased likelihood of fatal CVD and mortality [71]. These findings emphasize the critical importance of prioritizing the management of traditional CHD risk factors in RA patients as a pivotal step in reducing their overall CHD risk, which includes the risk of stroke [54, 72].

The emergence of evidence linking anti-CCP antibodies to cardiovascular risk, particularly in relation to stroke, underscores the significant overlap between these antibodies and cardiovascular health. Furthermore, the remarkable sensitivity of the anti-CCP2 antibody test, which enables detection years before clinical symptoms manifest, offers great potential as a predictive marker for early intervention and enhanced RA disease management, thereby minimizing the risk of cardiovascular disease, including stroke. However, further research is required to gain a deeper understanding of the association between stroke risk in RA patients exhibiting varying levels of CCP2 antibodies. This understanding could aid in predicting who is at risk of stroke, ultimately leading to improved management for individual RA patients.

## Multi-biomarker disease activity score for assessing stroke in RA patients

The treatment of RA is recommended to follow the “treat-to-target” strategy, which necessitates vigilant monitoring of disease activity. A valuable tool for this purpose is the Multi-Biomarker Disease Activity (MBDA) score system, which assesses serum levels of 12 specific biomarkers, including IL-6, TNF receptor type 1 (TNFR1), vascular cell adhesion molecule 1 (VCAM-1), epidermal growth factor (EGF), vascular endothelial growth factor A (VEGF-A), YKL-40, matrix metalloproteinase-1 (MMP-1), MMP-3, CRP, serum amyloid A (SAA), leptin, and resistin [73].

The MBDA test assesses RA disease activity by measuring these 12 serum protein biomarkers and subsequently generates a validated score that ranges from 1 to 100. This score is highly correlated with the Disease Activity Score in 28 joints with CRP (DAS28-CRP) [73]. The MBDA score serves as an objective and reliable system for monitoring disease progression. Consequently, it has the potential to significantly contribute to the development of personalized therapeutic plans that align with contemporary medical practices. In addition to its role in monitoring disease activity, the MBDA score also shows promise in predicting radiographic progression [74–77].

In 2019, the American College of Rheumatology Disease Activity Measures Working Group determined that the MBDA score met the minimum standards for regular use, ranking it among the top 11 measures for assessing RA disease activity [78]. Furthermore, independent research has shown that the MBDA score possesses predictive capabilities for future radiographic damage [79, 80]. In a comprehensive cross sectional observational study, the MBDA score demonstrated a significant association with the risk of cardiovascular disease (CVD) including stroke, suggesting that this score, along with some of its constituent biomarkers, can effectively detect inflammation relevant to cardiovascular pathology [81].

A new CVD risk score test based on MBDA has been developed to evaluate inflammation and predict the risk of major cardiovascular events (e.g., heart attack, stroke, or fatal CVD) in the next three years, specifically for RA patients [82]. This test is capable of factoring in the impact of RA inflammatory disease activity by incorporating both the MBDA score and three independent CVD-associated biomarkers. Notably, it outperformed prediction models that solely relied on clinical data [82].

The MBDA-based CVD risk prediction score offers rheumatologists a practical tool to assess CVD risk including stroke, thereby facilitating the management of traditional CVD risk factors and RA-related inflammation. To ensure the robustness of this predictive model, further validation is needed, involving extended time frames and more diverse cohorts of RA patients.

This MBDA-based CVD risk score test delivers actionable results at the point of care, underscoring the importance of mitigating systemic inflammation linked to RA. It can be considered as an adjunct to other tests for individuals aged 40 and above, especially those who are at high risk of cardiovascular diseases, such as stroke. Additionally, developing a focused MBDA-based assessment of stroke risk in association with RA disease activity has the potential to contribute to early stroke prevention and better management of RA patients, allowing for greater control over their associated risks.

## Linking the safety and efficacy of TNF-alpha inhibitors and JAK inhibitors in RA with stroke risk management

Tumor necrosis factor-alpha (TNF-alpha) inhibitors and Janus Kinase (JAK) inhibitors fall into two distinct categories of Disease-modifying anti-rheumatic drugs (DMARDs) frequently utilized in the management of RA [83, 84]. TNF-alpha, a pro-inflammatory cytokine produced by cells such as macrophages and monocytes during inflammatory responses, occupies a central position in the pro-inflammatory immune response [85]. In clinical practice, TNF inhibitors are employed to counteract elevated TNF levels that drive joint inflammation, thereby preventing tissue damage in RA.

The JAK family comprises receptor tyrosine kinases that, upon binding to specific cytokines, form dimers and subsequently phosphorylate signaling peptides belonging to the STAT family. These phosphorylated STAT proteins then migrate into the nucleus, where they govern the transcription of target genes. This signaling pathway ultimately regulates the expression of various cytokines, including IFN, IL-4, IL-6, and IL-10, all of which contribute to different immunological pathways and play roles in the pathogenesis of RA [86, 87].

The safety of these two categories of DMARDs was examined, with a particular emphasis on their use in RA patients at risk of cardiovascular diseases and their potential impact on various risk factors, including the occurrence of strokes. This evaluation was conducted as part of a prospective safety trial, known as the Oral Rheumatoid Arthritis Trial (ORAL) Surveillance, which was carried out during the development phase of tofacitinib, a JAK inhibitor. In this trial, the safety profile of tofacitinib was compared to that of TNF-alpha inhibitors [88, 89]. According to this study, the incidence of major cardiovascular (CV) events was higher in the tofacitinib group (3.4%) compared to the TNF-alpha inhibitors group (2.5%). The most common CV events observed were nonfatal myocardial infarctions in the tofacitinib group and nonfatal strokes in the TNF-alpha inhibitors group, especially among patients aged 65 years or older [90].

In a population-based study conducted in 2022, known as STAR-RA, Medicare data were analyzed for patients who had initiated treatment with either tofacitinib or TNF-alpha inhibitors. The study’s findings revealed no significant difference in the incidence rates of myocardial infarctions and strokes between these two treatment groups. This observation remained consistent even when the analysis was restricted to patients with similar cardiovascular risk factors as those in the ORAL Surveillance study [91].

Furthermore, a meta-analysis revealed that RA patients treated with TNF-alpha inhibitors experienced a 30% reduction in the risk of CV events [92]. Specifically, among studies focusing on myocardial infarctions or strokes as outcomes, the use of TNF-alpha inhibitors was associated with a 41% relative risk reduction in myocardial infarctions and a 43% reduction in strokes. Notably, these effects may not be universal among all patients receiving TNF-alpha inhibitors but appear to be related to clinical response. Some studies have observed a reduction in CV events only in patients who respond well to anti-TNF-alpha treatment [93, 94]. TNF-alpha inhibitors may achieve this effect by improving endothelial dysfunction and reducing oxidative stress.

These findings underscore the significance of customizing RA treatment strategies to individual patient profiles, highlighting the imperative for a personalized approach in managing their complex autoimmune disease, considering their specific cardiovascular risk factors, including stroke.

## Rheumatoid factor and IL-6 inhibition in ischemic stroke: current and future perspectives

Rheumatoid factor (RF) is an autoantibody frequently found in the blood of individuals with RA. It targets the body’s own proteins, leading to the formation of immune complexes (ICs) and triggering an immune response. RF serves as a well-established diagnostic biomarker for RA, which has the high potential to confirm the presence of the disease [95, 96]. However, the relationship between RF and the prognosis and treatment of ischemic stroke is still not extensively studied. RF has been associated with cardiovascular diseases [97, 98], immune complexes (ICs) and complement-mediated inflammation [99], as well as endothelial dysfunction [29]. Positive RF status has been linked to increased risks and poor prognosis of cardiovascular diseases [53, 100–104]. However, the specific effects of RF on clinical outcomes in ischemic stroke are not well understood.

The existing literature on the relationship between RF and stroke is limited, with only a few studies having investigated this association. One study discovered a positive correlation between elevated serum RF levels and cognitive impairment three months after an ischemic stroke in patients. This suggests that higher levels of RF may be linked to cognitive difficulties following a stroke [105]. Another study examined stroke patients with RA who tested positive for RF and found an increased mortality rate among this group. However, it is important to note that the statistical power of both these studies was limited due to their small sample size [12].

The identification of specific genes and risk loci associated with RA has been a successful endeavor in genetic research. Scientists have located these genes and risk loci on chromosomes, and one key factor involved in RA is interleukin 6 (IL-6), a cytokine responsible for the inflammatory response. This discovery prompted researchers to investigate the relationship between IL-6 and ischemic stroke. A comprehensive genome-wide association study was conducted to explore the connection between IL-6 and ischemic stroke [106–108]. The study revealed that individuals with a genetic predisposition to RA face an increased risk of experiencing negative outcomes following an ischemic stroke, indicating a genetic susceptibility. Conversely, individuals who possess genetic traits that predict inhibition of IL-6 demonstrate a reduced risk of adverse outcomes in such cases. Moreover, the presence of positive RF at the baseline is associated with a heightened risk of death or significant disability after a stroke. However, individuals who have genetically predicted IL-6 inhibition exhibit a lower risk of adverse outcomes in similar circumstances [109].

The effects of IL-6, along with RF and RA, on the prognosis, early treatment, and prevention of ischemic stroke are not well defined. However, there are several proposed biological mechanisms that could explain the potential relationship between these factors and ischemic stroke. IL-6, RF, and RA may contribute to the activation of the complement system, a part of the immune system that plays a role in inflammation and immune response, which can lead to increased inflammation and potentially impact the development or progression of ischemic stroke [99]. They might also contribute to damage or dysfunction of the vascular endothelium, which can disrupt normal blood flow and increase the risk of developing ischemic stroke [102]. Additionally, they may be associated with increased oxidative stress in the body which can promote inflammation and contribute to the development or progression of ischemic stroke [110]. These factors might also play a role in driving the progression of atherosclerosis, leading to the formation of plaques. Consequently, these plaques can increase the risk of ischemic stroke by impeding blood flow or by promoting the formation of blood clots that have the potential to block arteries [103, 111]. Furthermore, there have been indications that IL-6 may contribute to the disruption of cerebrovascular regulation and heightened tissue damage following a stroke [112]. Therefore, IL-6 inhibition therapy may have a beneficial effect on the prognosis of ischemic stroke patients with RA by reducing leptin levels and counteracting harmful effects on neuron formation and nerve stem cell proliferation [113, 114]. The expression of endothelial tissue factor can be upregulated by IL-6, resulting in the development of a prothrombotic state [115]. In addition, IL-20, another member of the Interleukin family, is associated with rheumatoid arthritis and has the ability to stimulate the production of IL-6, which could potentially contribute to the onset of stroke. (RA) [116, 117].

Previous studies have explored the relationship between RA, IL-6 inhibition, and stroke prognosis, suggesting that IL-6 inhibitors could be a promising treatment option for improving the prognosis of ischemic stroke patients with RA [109, 114, 118, 119]. However, further research is necessary to establish the clinical benefits of IL-6 inhibitors in enhancing the prognosis of RA patients with ischemic stroke.

In the acute phase of atherosclerotic ischemic stroke, it is crucial to test for RF. Early intervention with optimal adjunctive medical therapy is crucial for patients who test positive for RF, particularly those diagnosed with RA. It helps mitigate the risk of adverse outcomes associated with the condition. However, it is important to highlight that certain NSAID agents may potentially accelerate the progression of stroke [5, 120]. Therefore, caution should be exercised when considering treatment options.

To better understand the relationship between RF, RA, and the prognosis and treatment of ischemic stroke, further extensive investigation is necessary. This would involve conducting studies and research to investigate the mechanisms through which RF and RA influence the development and prognosis of ischemic stroke. By unraveling these mechanisms, researchers can gain insights into the underlying processes that contribute to stroke risk in individuals with RA. Furthermore, understanding the relationship between RF, RA, and ischemic stroke may have significant implications, enabling the identification of specific biomarkers or indicators that could help identify individuals with rheumatoid arthritis who are at a higher risk of developing ischemic stroke. Additionally, it may lead to the development of targeted treatment strategies to reduce this risk and improve outcomes for individuals with both RA and ischemic stroke.

## The association between NSAIDs and risk of ischemic stroke: a review of conflicting evidence

The utilization of nonsteroidal anti-inflammatory drugs (NSAIDs) in patients with RA and its potential correlation with the risk of ischemic stroke has been a subject of concern. Nevertheless, the current body of literature regarding the use of NSAIDs and its relationship to the risk of ischemic stroke has yielded conflicting results. Some studies have pointed to an elevated risk of stroke associated with specific NSAIDs; while, others have proposed a neutral or potentially protective effect.

Several studies have examined the impact of specific NSAIDs such as Rofecoxib, Celecoxib and Diclofenac on the risk of stroke. However, these studies have reported mixed results, with some indicating an increased risk of stroke associated with the use of these drugs [121–127], while others found no significant effect or even a potential protective effect [128–131]. For example, a comprehensive review of 75 observational studies concluded that the use of Rofecoxib and Diclofenac is associated with an increased risk of stroke [132]. Similarly, a study conducted in Australia demonstrated that the use of any NSAID was associated with a 1.88 times higher risk of ischemic stroke [133]. Other studies have also linked specific NSAIDs like Indomethacin, Rofecoxib, and Celecoxib to an elevated risk of stroke [121, 127, 133–136].

On the contrary, other studies have reported contrasting results. A meta-analysis investigating the correlation between cyclooxygenase-2 (COX-2) selectivity and cardiovascular risk found that, except for Rofecoxib, other NSAIDs and COX-2 inhibitors did not show a statistically significant correlation with cardiovascular death [128–131]. In fact, Celecoxib was found to decrease the incidence rate of cardiovascular events. Additionally, a large patient-level meta-analysis concluded that there was no difference in cardiovascular events between Celecoxib and non-selective NSAIDs [137].

NSAIDs have important considerations regarding their impact on cardiovascular health. While they can contribute to a higher risk of atherosclerosis and stroke [132, 138, 139], it is crucial to understand the underlying mechanisms behind this increased risk. The increased risk of atherosclerosis and stroke associated with NSAIDs is attributed to their effects on cardiovascular function, platelet aggregation, and smooth muscle proliferation by altering the balance of prostaglandins and thromboxane [123, 140, 141]. However, NSAIDs also have a multifaceted impact on cardiovascular health due to their ability to inhibit COX enzymes. These enzymes play a role in the production of prostaglandins. By inhibiting COX enzymes, NSAIDs reduce the production of prostaglandins, which leads to a decrease in inflammation, pain, smooth muscle proliferation, and platelet aggregation [142–146]. This reduction in inflammatory processes and platelet aggregation can have positive effects on cardiovascular health. Furthermore, NSAIDs inhibit thromboxane, which is a vasoconstrictor. By suppressing thromboxane synthesis, NSAIDs help to dilate blood vessels, reducing the likelihood of blockages and thereby lowering the risk of ischemic stroke [141, 143, 147–151].

Long-term use of Celecoxib may gradually lead to adverse effects such as increased blood pressure and a potentially prothrombotic environment, which can elevate the relative risk of ischemic stroke [152]. A population-based retrospective cohort study suggests that Celecoxib and Etoricoxib may be associated with a reduced risk of first-occurrence ischemic stroke in RA patients, with the risk reduction dependent on the dose and duration of use [153]. These paradoxical results of Celecoxib being protective at a higher dose in a shorter duration but harmful in a longer duration, as well as the neutral to slight protective effects of Etoricoxib, require further investigation to understand their underlying physiological mechanisms. Clinical trials mentioned in a patient-level meta-analysis concluded that there was no difference in the incidence of cardiovascular events between Celecoxib and non-selective NSAIDs [137, 154]. Additional clinical trials may be necessary to establish causality rather than just an association between NSAID use and the risk of ischemic stroke. It is important to note that these findings are based on observational studies, which have inherent limitations and may be subject to confounding factors. The conflicting results in the literature could be attributed to differences in study design, populations studied, duration of NSAID use, dosages, and other factors that may influence the risk of stroke.

Many women use NSAIDs for relief from dysmenorrhea, potentially exposing themselves to an elevated risk of ischemic stroke, particularly among young females. This risk increases with prolonged medication usage [155]. Hence, it is crucial that every woman displaying symptoms of dysmenorrhea receives specialized outpatient treatment and care.

The risk of ischemic stroke varies across different NSAIDs and seems to be elevated in individuals with a prior history of ischemic stroke or transient ischemic attack (TIA), as well as in younger or male patients. Co-administration of aspirin, other antiplatelets, or anticoagulants may potentially reduce this risk. The observed modest to moderate increase in risk of ischemic stroke (ranging from 13 to 46%) associated with NSAID use raises public health concerns, given the widespread usage of these medications [126].

Hence, although there is some evidence indicating a potential higher risk of ischemic stroke linked to specific NSAIDs in individuals with rheumatoid arthritis (RA), additional research is necessary to establish this connection conclusively. Future studies should address the limitations of previous research and offer more definitive conclusions regarding the safety of NSAID usage in this patient group. In the interim, healthcare providers should thoroughly assess the potential risks and benefits of NSAID therapy on a case-by-case basis when treating RA patients, particularly those at an elevated risk of stroke and other cardiovascular complications.

## Substance P in RA: insights into inflammatory mechanisms and preventative perspectives on stroke

In clinical cases of ischemic stroke, elevated levels of substance P (SP) have been documented in the serum of human patients with complete stroke, transient ischemic attack (TIA), as well as in studies involving animal stroke models [156, 157]. This neuropeptide, associated with various inflammatory processes, suggests its involvement in the pathogenesis of inflammatory arthritis, such as RA. Studies have revealed increased SP levels in the synovial fluid and serum of RA patients [158]. As a neuroinflammatory mediator, SP is produced by sensory nerve fibers and local inflammatory cells, playing a significant role in the skeletal degeneration and damage induced by chronic inflammation [159, 160]. Anomalies in the SP-NK1R pathway, where NK1R (receptors with the highest affinity for SP) is involved, have been observed in various inflammatory diseases, indicating its potential role in inflammatory processes [161, 162]. Consequently, suppressing pro-inflammatory effects through NK1R antagonists might offer a therapeutic avenue for RA patients and potentially act preventatively against stroke occurrences.

Moreover, studies have indicated the potential use of NK1R antagonists, such as aprepitant, in RA. Aprepitant has shown decreased release of inflammatory factors in fibroblast-like synoviocytes in RA [163]. Blocking NK1R may present a novel therapeutic approach for autoimmune-related inflammatory diseases like RA and other inflammatory arthritis. However, it remains unclear whether this NK1R antagonist can effectively mitigate disease activity in RA, given its various clinical manifestations, potentially acting preventatively against stroke. Additionally, serum SP levels have been identified as an indicator of disease activity and subclinical inflammation in RA patients [164]. It has also been suggested that SP might be linked to the suppression and blockade of inflammatory responses in RA [165]. Interestingly, both SP and NK1R antagonists have exhibited similar anti-inflammatory effects.

Notably, SP demonstrates an anti-inflammatory function by increasing IL-10 and decreasing TNF-α [166]. These seemingly contradictory results hint at the SP-NK1R pathway playing a role as an immune modulator rather than an excessive expressor in the pathogenesis of inflammatory diseases. It is essential to note that anti-TNF therapy using etanercept has been shown to lower substance P levels. Patients who did not experience this reduction did not benefit from the treatment [167]. Further in-depth research to unveil the specific mechanisms related to the SP-NK1R pathway is crucial, given its significant role in RA treatment and the potential prevention of stroke.

One of the fascinating clinical observations in patients with RA has been that individuals who have experienced hemiplegia, a condition caused by brain damage, such as in the case of a stroke, resulting in paralysis on one side of the body, do not exhibit joint involvement on the hemiplegic side when they later develop RA [168, 169]. While a simplistic explanation could attribute this to a lack of movement, it appears more probable that this phenomenon is a result of a crucial interaction between the disease process and the nervous system. Specifically, SP levels have been found to be elevated in patients with RA, particularly within the joints [170]. Consequently, there is an urgent need for future studies to investigate the role of SP in RA patients and its potential correlation with the risk of hemiplegia caused by post-stroke conditions.

## Discussion

Several research studies have investigated the relationship between stroke and RA. RA, characterized by chronic inflammation, shows a heightened association with an increased risk of stroke [13, 38, 171]. Notably, this risk is independent of traditional cardiovascular risk factors [41] and is particularly prominent in younger individuals with RA [172]. The pathogenesis of stroke in RA involves shared inflammatory and immune mediators that affect blood vessels in the brain [173]. Inflammatory cytokines released in the joints can enter the bloodstream and contribute to atherosclerosis and stroke [174]. RA can also lead to cardioembolic stroke through nervous system vasculitis and cardiac complications [26]. Managing the risk of stroke in RA patients involves focusing on cardiovascular risk management, including lifestyle changes such as a nutritious diet, regular physical activity, and quitting smoking [175].

While the link between RA and the risk of stroke has been firmly established, it is worth noting that some studies have not demonstrated an increased risk of stroke in individuals with RA. This lack of association could be attributed to variations in the definitions of stroke used in various studies. Notably, despite clear distinctions in risk factor profiles and the differing pathogenesis of ischemic and hemorrhagic strokes, certain studies have chosen to use composite outcomes that encompass both types of strokes [176, 177]. Given the fundamental disparities between ischemic and hemorrhagic strokes, it is imperative to classify stroke types separately and assess the risk for these two distinct entities rather than relying on a composite outcome.

The involvement of the anti-CCP response in the pathogenesis of RA may be attributed to the established association between anti-CCP antibodies and the severity of RA. Additionally, numerous studies have indicated a link between anti-citrullinated protein antibodies (ACPAs) and an elevated risk of acute myocardial infarction, major adverse cardiovascular events, and stroke [178–180]. Consequently, an approach could be employed to effectively reduce the risk of stroke and other cardiovascular complications in individuals with RA. This approach encompasses monitoring anti-CCP antibodies, addressing conventional cardiovascular risk factors, and managing inflammation. Healthcare providers could potentially enhance cardiovascular well-being and overall quality of life for RA patients, ultimately reducing the burden of cardiovascular diseases, including stroke.

The Multi-Biomarker Disease Activity (MBDA) score holds significant value in the care of RA patients, serving as a valuable tool for both monitoring disease activity and predicting radiological progression [181]. Nevertheless, further research is required to more effectively assess the utility of the MBDA score and explore the potential role of individual biomarkers in monitoring disease activity, especially in the context of cardiovascular diseases such as stroke in association with RA. While several studies have examined the usefulness of the MBDA score, and a meta-analysis has investigated its correlation with conventional disease activity measures (DAMs), a comprehensive analysis of its predictive and discriminative capabilities has not yet been undertaken [182].

The recent development and validation of a CVD risk prediction score tailored specifically for RA patients involves the integration of routine clinical assessments and RA-related biomarkers to predict the risk of CVD, including stroke [82]. This innovative approach aims to enhance preventive CVD care for individuals with RA by constructing a prognostic score that incorporates the impact of RA-related inflammation on an individual’s CVD risk. The primary objective of this initiative is to establish a validated CVD risk scoring system that allows rheumatologists to effectively assess the risk of CVD in their RA patients during routine office visits. This scoring system will incorporate RA disease activity indicators to provide a more comprehensive estimate of cardiovascular risk, including factors related to stroke.

The association between RF and stroke prognosis and treatment requires further investigation. RF has been linked to increased risks and poor prognosis of cardiovascular diseases [183], but its impact on ischemic stroke outcomes is not well understood. Some studies suggest potential benefits of IL-6 inhibition in improving stroke prognosis in RA patients, but more research is needed [109, 119]. By examining specific blood proteins associated with both blood clotting and inflammation, researchers hope to identify ways to predict the risk of stroke. This knowledge could contribute to primary and secondary prevention of stroke and the development of new stroke prevention drugs, which is particularly important for younger adults with inflammatory diseases.

Systemic inflammation in individuals diagnosed with RA not only accelerates the progression of atherosclerosis but also induces a prothrombotic state, increasing the susceptibility to cardiovascular (CV) events, including stroke [184]. As a result, DMARDs, through their anti-inflammatory properties, possess the capacity to intervene at various stages of the CV event pathway. Consequently, they could potentially play a pivotal role in mitigating the stroke risk in RA patients [185].

TNF inhibitors and JAK inhibitors are both employed in the treatment of rheumatoid RA by targeting distinct immune pathways. Their safety profiles with respect to cardiovascular events including stroke may differ, as indicated by some studies, which propose potential variations possibly influenced by individual patient responses to treatment. Therefore, it is imperative to conduct further research and take into account individual patient characteristics when determining the most suitable DMARD therapy for RA.

The use of NSAIDs in RA patients and their association with the risk of ischemic stroke have yielded conflicting findings. To arrive at more conclusive and definitive conclusions regarding the safety of NSAID use in RA patients, there is a pressing need for further well-designed and well-controlled research. Analyzing the association between NSAID medication use and the risk factors for cardiovascular disease (CVD), particularly stroke, is imperative for individuals with RA. This is because NSAIDs, while effective at reducing inflammation, can potentially have adverse effects on CVD, especially ischemic stroke. These adverse effects can lead to significant disabilities in patients, underscoring the importance of enhancing treatment management and expanding the available treatment options.

In conclusion, a comprehensive analysis of risk factors and associations between stroke and RA emphasizes the need for targeted healthcare practices for individuals with RA. Early detection, management of RA severity, control of cardiovascular risk factors, and further research into treatment options are essential for reducing the risk of stroke and improving outcomes for individuals with both RA and ischemic stroke.

## Data Availability

Not applicable.

## References

[1] Hankey GJ. Stroke. Lancet. 2017;389(10069):641–54. 27637676 10.1016/S0140-6736(16)30962-X

[2] Collaborators GBDS. Global, regional, and national burden of stroke and its risk factors, 1990–2019: a systematic analysis for the Global Burden of Disease Study 2019. Lancet Neurol. 2021;20(10):795–820. 34487721 10.1016/S1474-4422(21)00252-0 PMC8443449

[3] Feigin VL, et al. The global burden of neurological disorders: translating evidence into policy. Lancet Neurol. 2020;19(3):255–65. 31813850 10.1016/S1474-4422(19)30411-9 PMC9945815

[4] Guo Q, et al. Rheumatoid arthritis: pathological mechanisms and modern pharmacologic therapies. Bone Res. 2018;6:15. 29736302 10.1038/s41413-018-0016-9 PMC5920070

[5] Nadareishvili Z, et al. Cardiovascular, rheumatologic, and pharmacologic predictors of stroke in patients with rheumatoid arthritis: a nested, case-control study. Arthritis Rheum. 2008;59(8):1090–6. 18668583 10.1002/art.23935 PMC2778069

[6] Sherer Y, Shoenfeld Y. Mechanisms of disease: atherosclerosis in autoimmune diseases. Nat Clin Pract Rheumatol. 2006;2(2):99–106. 16932663 10.1038/ncprheum0092

[7] del Rincon ID, et al. High incidence of cardiovascular events in a rheumatoid arthritis cohort not explained by traditional cardiac risk factors. Arthritis Rheum. 2001;44(12):2737–45. 11762933 10.1002/1529-0131(200112)44:12<2737::AID-ART460>3.0.CO;2-%23

[8] Kremers HM, et al. High ten-year risk of cardiovascular disease in newly diagnosed rheumatoid arthritis patients: a population-based cohort study. Arthritis Rheum. 2008;58(8):2268–74. 18668561 10.1002/art.23650 PMC2929699

[9] Solomon DH, et al. Patterns of cardiovascular risk in rheumatoid arthritis. Ann Rheum Dis. 2006;65(12):1608–12. 16793844 10.1136/ard.2005.050377 PMC1798453

[10] Masuda H, et al. Disease duration and severity impacts on long-term cardiovascular events in Japanese patients with rheumatoid arthritis. J Cardiol. 2014;64(5):366–70. 24685688 10.1016/j.jjcc.2014.02.018

[11] Semb AG, et al. Lipids, myocardial infarction and ischaemic stroke in patients with rheumatoid arthritis in the Apolipoprotein-related Mortality RISk (AMORIS) Study. Ann Rheum Dis. 2010;69(11):1996–2001. 20551156 10.1136/ard.2009.126128

[12] Sodergren A, et al. Increased incidence of stroke and impaired prognosis after stroke among patients with seropositive rheumatoid arthritis. Clin Exp Rheumatol. 2009;27(4):641–4. 19772797

[13] Liou TH, et al. Risk of stroke in patients with rheumatism: a nationwide longitudinal population-based study. Sci Rep. 2014;4:5110. 24898360 10.1038/srep05110 PMC4046260

[14] Watts RA, et al. Rheumatoid vasculitis: Becoming extinct? Rheumatology (Oxford). 2004;43(7):920–3. 15126674 10.1093/rheumatology/keh210

[15] Agarwal A, Bathla G, Kanekar S. Imaging of non-atherosclerotic vasculopathies. J Clin Imaging Sci. 2020;10:62. 33194304 10.25259/JCIS_91_2020 PMC7656038

[16] Dhillon N, Liang K. Prevention of stroke in rheumatoid arthritis. Curr Treat Options Neurol. 2015;17(7):356. 25981388 10.1007/s11940-015-0356-3

[17] Damasio MB, et al. Synovial and inflammatory diseases in childhood: role of new imaging modalities in the assessment of patients with juvenile idiopathic arthritis. Pediatr Radiol. 2010;40(6):985–98. 20432018 10.1007/s00247-010-1612-z

[18] Goronzy JJ, Weyand CM. Developments in the scientific understanding of rheumatoid arthritis. Arthritis Res Ther. 2009;11(5):249. 19835638 10.1186/ar2758 PMC2787299

[19] Gonzalez-Gay MA, Gonzalez-Juanatey C, Martin J. Rheumatoid arthritis: a disease associated with accelerated atherogenesis. Semin Arthritis Rheum. 2005;35(1):8–17. 16084219 10.1016/j.semarthrit.2005.03.004

[20] van Leuven SI, et al. Systemic inflammation as a risk factor for atherothrombosis. Rheumatology (Oxford). 2008;47(1):3–7. 17702769 10.1093/rheumatology/kem202

[21] Gonzalez-Gay MA, et al. High-grade C-reactive protein elevation correlates with accelerated atherogenesis in patients with rheumatoid arthritis. J Rheumatol. 2005;32(7):1219–23. 15996055

[22] Wick G, Knoflach M, Xu Q. Autoimmune and inflammatory mechanisms in atherosclerosis. Annu Rev Immunol. 2004;22:361–403. 15032582 10.1146/annurev.immunol.22.012703.104644

[23] Miller RE, Miller RJ, Malfait AM. Osteoarthritis joint pain: the cytokine connection. Cytokine. 2014;70(2):185–93. 25066335 10.1016/j.cyto.2014.06.019 PMC4254338

[24] Libby P. Molecular and cellular mechanisms of the thrombotic complications of atherosclerosis. J Lipid Res. 2009;50(Suppl):S352-357. 19096046 10.1194/jlr.R800099-JLR200 PMC2674742

[25] Zaman AG, et al. The role of plaque rupture and thrombosis in coronary artery disease. Atherosclerosis. 2000;149(2):251–66. 10729375 10.1016/s0021-9150(99)00479-7

[26] Rawla P. Cardiac and vascular complications in rheumatoid arthritis. Reumatologia. 2019;57(1):27–36. 30858628 10.5114/reum.2019.83236 PMC6409824

[27] Lindhardsen J, et al. Risk of atrial fibrillation and stroke in rheumatoid arthritis: Danish nationwide cohort study. BMJ. 2012;344: e1257. 22403267 10.1136/bmj.e1257 PMC3297675

[28] Ross R. Atherosclerosis—an inflammatory disease. N Engl J Med. 1999;340(2):115–26. 9887164 10.1056/NEJM199901143400207

[29] Abou-Raya A, Abou-Raya S. Inflammation: a pivotal link between autoimmune diseases and atherosclerosis. Autoimmun Rev. 2006;5(5):331–7. 16782558 10.1016/j.autrev.2005.12.006

[30] Wang J, et al. A Role of IL-17 in rheumatoid arthritis patients complicated with atherosclerosis. Front Pharmacol. 2022;13: 828933. 35211020 10.3389/fphar.2022.828933 PMC8861488

[31] Hallenbeck JM, Hansson GK, Becker KJ. Immunology of ischemic vascular disease: plaque to attack. Trends Immunol. 2005;26(10):550–6. 16109503 10.1016/j.it.2005.08.007

[32] Lima DS, et al. Brachial endothelial function is impaired in patients with systemic lupus erythematosus. J Rheumatol. 2002;29(2):292–7. 11842823

[33] Vaudo G, et al. Endothelial dysfunction in young patients with rheumatoid arthritis and low disease activity. Ann Rheum Dis. 2004;63(1):31–5. 14672888 10.1136/ard.2003.007740 PMC1754717

[34] Akhmedov A, et al. TNFalpha induces endothelial dysfunction in rheumatoid arthritis via LOX-1 and arginase 2: reversal by monoclonal TNFalpha antibodies. Cardiovasc Res. 2022;118(1):254–66. 33483748 10.1093/cvr/cvab005

[35] Suss P, et al. The joint-brain axis: insights from rheumatoid arthritis on the crosstalk between chronic peripheral inflammation and the brain. Front Immunol. 2020;11: 612104. 33362800 10.3389/fimmu.2020.612104 PMC7758283

[36] Tam LH, et al. Effect of treat-to-target strategies aiming at remission of arterial stiffness in early rheumatoid arthritis: a randomized controlled study. J Rheumatol. 2018;45(9):1229–39. 29764965 10.3899/jrheum.171128

[37] Nishioku T, et al. Potential role for S100A4 in the disruption of the blood-brain barrier in collagen-induced arthritic mice, an animal model of rheumatoid arthritis. Neuroscience. 2011;189:286–92. 21627981 10.1016/j.neuroscience.2011.05.044

[38] Liu W, et al. Stroke risk in arthritis: a systematic review and meta-analysis of cohort studies. PLoS ONE. 2021;16(3): e0248564. 33725018 10.1371/journal.pone.0248564 PMC7963101

[39] Trommer K, et al. Increased incidence of stroke and transient ischemic attack in patients with rheumatoid arthritis and ankylosing spondylitis in Germany. Neuroepidemiology. 2021;55(2):162–70. 33789293 10.1159/000514889

[40] Boden-Albala B, Sacco RL. Lifestyle factors and stroke risk: exercise, alcohol, diet, obesity, smoking, drug use, and stress. Curr Atheroscler Rep. 2000;2(2):160–6. 11122740 10.1007/s11883-000-0111-3

[41] Boehme AK, Esenwa C, Elkind MS. Stroke risk factors, genetics, and prevention. Circ Res. 2017;120(3):472–95. 28154098 10.1161/CIRCRESAHA.116.308398 PMC5321635

[42] He FJ, Nowson CA, MacGregor GA. Fruit and vegetable consumption and stroke: meta-analysis of cohort studies. Lancet. 2006;367(9507):320–6. 16443039 10.1016/S0140-6736(06)68069-0

[43] Hu X, et al. Migraine and the risk of stroke: an updated meta-analysis of prospective cohort studies. Neurol Sci. 2017;38(1):33–40. 27785579 10.1007/s10072-016-2746-z

[44] Li W, et al. Sleep duration and risk of stroke events and stroke mortality: a systematic review and meta-analysis of prospective cohort studies. Int J Cardiol. 2016;223:870–6. 27584562 10.1016/j.ijcard.2016.08.302

[45] Li Z, et al. Anemia increases the mortality risk in patients with stroke: a meta-analysis of cohort studies. Sci Rep. 2016;6:26636. 27211606 10.1038/srep26636 PMC4876389

[46] Yuan M, et al. Inflammatory bowel disease and risk of stroke: a meta-analysis of cohort studies. Int J Cardiol. 2016;202:106–9. 26386936 10.1016/j.ijcard.2015.08.190

[47] Parikh NS, Merkler AE, Iadecola C. Inflammation, autoimmunity, infection, and stroke: epidemiology and lessons from therapeutic intervention. Stroke. 2020;51(3):711–8. 32078460 10.1161/STROKEAHA.119.024157 PMC7041866

[48] Agca R, et al. EULAR recommendations for cardiovascular disease risk management in patients with rheumatoid arthritis and other forms of inflammatory joint disorders: 2015/2016 update. Ann Rheum Dis. 2017;76(1):17–28. 27697765 10.1136/annrheumdis-2016-209775

[49] Bonek K, Gluszko P. Cardiovascular risk assessment in rheumatoid arthritis—controversies and the new approach. Reumatologia. 2016;54(3):128–35. 27504023 10.5114/reum.2016.61214 PMC4967980

[50] Wiseman SJ, Ralston SH, Wardlaw JM. Cerebrovascular disease in rheumatic diseases: a systematic review and meta-analysis. Stroke. 2016;47(4):943–50. 26917565 10.1161/STROKEAHA.115.012052

[51] Fransen J, et al. Rheumatoid arthritis disadvantages younger patients for cardiovascular diseases: a meta-analysis. PLoS ONE. 2016;11(6): e0157360. 27310259 10.1371/journal.pone.0157360 PMC4911000

[52] Avina-Zubieta JA, et al. Risk of incident cardiovascular events in patients with rheumatoid arthritis: a meta-analysis of observational studies. Ann Rheum Dis. 2012;71(9):1524–9. 22425941 10.1136/annrheumdis-2011-200726

[53] Lee DH, et al. Association between ischemic stroke and seropositive rheumatoid arthritis in Korea: a nationwide longitudinal cohort study. PLoS ONE. 2021;16(5): e0251851. 33999944 10.1371/journal.pone.0251851 PMC8128246

[54] Solomon DH, et al. Explaining the cardiovascular risk associated with rheumatoid arthritis: traditional risk factors versus markers of rheumatoid arthritis severity. Ann Rheum Dis. 2010;69(11):1920–5. 20444756 10.1136/ard.2009.122226 PMC2963658

[55] Solomon DH, et al. Disease activity in rheumatoid arthritis and the risk of cardiovascular events. Arthritis Rheumatol. 2015;67(6):1449–55. 25776112 10.1002/art.39098 PMC4446181

[56] Micha R, et al. Systematic review and meta-analysis of methotrexate use and risk of cardiovascular disease. Am J Cardiol. 2011;108(9):1362–70. 21855836 10.1016/j.amjcard.2011.06.054 PMC3196048

[57] Barnabe C, Martin BJ, Ghali WA. Systematic review and meta-analysis: anti-tumor necrosis factor alpha therapy and cardiovascular events in rheumatoid arthritis. Arthritis Care Res (Hoboken). 2011;63(4):522–9. 20957658 10.1002/acr.20371

[58] Kang S, et al. associations between cardiovascular outcomes and rheumatoid arthritis: a nationwide population-based cohort study. J Clin Med. 2022;11(22):6812. 36431290 10.3390/jcm11226812 PMC9695475

[59] van Venrooij WJ, van Beers JJ, Pruijn GJ. Anti-CCP antibodies: the past, the present and the future. Nat Rev Rheumatol. 2011;7(7):391–8. 21647203 10.1038/nrrheum.2011.76

[60] Barbarroja N, et al. Anticyclic citrullinated protein antibodies are implicated in the development of cardiovascular disease in rheumatoid arthritis. Arter Thromb Vasc Biol. 2014;34(12):2706–16. 10.1161/ATVBAHA.114.304475 25256232

[61] Taylor P, et al. A systematic review of serum biomarkers anti-cyclic citrullinated Peptide and rheumatoid factor as tests for rheumatoid arthritis. Autoimmune Dis. 2011;2011: 815038. 21915375 10.4061/2011/815038 PMC3170888

[62] Bukhari M, et al. The performance of anti-cyclic citrullinated peptide antibodies in predicting the severity of radiologic damage in inflammatory polyarthritis: results from the Norfolk Arthritis Register. Arthritis Rheum. 2007;56(9):2929–35. 17763407 10.1002/art.22868 PMC2435419

[63] Ronnelid J, et al. Longitudinal analysis of citrullinated protein/peptide antibodies (anti-CP) during 5 year follow up in early rheumatoid arthritis: anti-CP status predicts worse disease activity and greater radiological progression. Ann Rheum Dis. 2005;64(12):1744–9. 15843452 10.1136/ard.2004.033571 PMC1755292

[64] Gerli R, et al. Association of anti-cyclic citrullinated peptide antibodies with subclinical atherosclerosis in patients with rheumatoid arthritis. Ann Rheum Dis. 2008;67(5):724–5. 18408112 10.1136/ard.2007.073718

[65] Rantapaa-Dahlqvist S, et al. Antibodies against cyclic citrullinated peptide and IgA rheumatoid factor predict the development of rheumatoid arthritis. Arthritis Rheum. 2003;48(10):2741–9. 14558078 10.1002/art.11223

[66] Bartoloni E, et al. Diagnostic value of anti-mutated citrullinated vimentin in comparison to anti-cyclic citrullinated peptide and anti-viral citrullinated peptide 2 antibodies in rheumatoid arthritis: an Italian multicentric study and review of the literature. Autoimmun Rev. 2012;11(11):815–20. 22394488 10.1016/j.autrev.2012.02.015

[67] Aletaha D, et al. 2010 Rheumatoid arthritis classification criteria: an American College of Rheumatology/European League Against Rheumatism collaborative initiative. Arthritis Rheum. 2010;62(9):2569–81. 20872595 10.1002/art.27584

[68] Nishimura K, et al. Meta-analysis: diagnostic accuracy of anti-cyclic citrullinated peptide antibody and rheumatoid factor for rheumatoid arthritis. Ann Intern Med. 2007;146(11):797–808. 17548411 10.7326/0003-4819-146-11-200706050-00008

[69] Bizzaro N, et al. Analytical and diagnostic characteristics of 11 2nd- and 3rd-generation immunoenzymatic methods for the detection of antibodies to citrullinated proteins. Clin Chem. 2007;53(8):1527–33. 17586589 10.1373/clinchem.2007.087569

[70] van der Helm-van Mil AH, et al. The HLA-DRB1 shared epitope alleles are primarily a risk factor for anti-cyclic citrullinated peptide antibodies and are not an independent risk factor for development of rheumatoid arthritis. Arthritis Rheum. 2006;54(4):1117–21. 16572446 10.1002/art.21739

[71] Mackey RH, et al. Rheumatoid arthritis, anti-cyclic citrullinated peptide positivity, and cardiovascular disease risk in the women’s health initiative. Arthritis Rheumatol. 2015;67(9):2311–22. 25988241 10.1002/art.39198 PMC4551571

[72] Gomez-Vaquero C, et al. Assessment of cardiovascular risk in rheumatoid arthritis: impact of the new EULAR recommendations on the score cardiovascular risk index. Clin Rheumatol. 2012;31(1):35–9. 21567119 10.1007/s10067-011-1774-6

[73] Curtis JR, et al. Validation of a novel multibiomarker test to assess rheumatoid arthritis disease activity. Arthritis Care Res (Hoboken). 2012;64(12):1794–803. 22736476 10.1002/acr.21767 PMC3508159

[74] Bakker MF, et al. Performance of a multi-biomarker score measuring rheumatoid arthritis disease activity in the CAMERA tight control study. Ann Rheum Dis. 2012;71(10):1692–7. 22596166 10.1136/annrheumdis-2011-200963 PMC3439649

[75] Brahe CH, et al. Predictive value of a multi-biomarker disease activity score for clinical remission and radiographic progression in patients with early rheumatoid arthritis: a post-hoc study of the OPERA trial. Scand J Rheumatol. 2019;48(1):9–16. 29985080 10.1080/03009742.2018.1464206

[76] Hambardzumyan K, et al. Pretreatment multi-biomarker disease activity score and radiographic progression in early RA: results from the SWEFOT trial. Ann Rheum Dis. 2015;74(6):1102–9. 24812287 10.1136/annrheumdis-2013-204986 PMC4431327

[77] Markusse IM, et al. A multibiomarker disease activity score for rheumatoid arthritis predicts radiographic joint damage in the BeSt study. J Rheumatol. 2014;41(11):2114–9. 25128518 10.3899/jrheum.131412

[78] England BR, et al. 2019 update of the American college of rheumatology recommended rheumatoid arthritis disease activity measures. Arthritis Care Res (Hoboken). 2019;71(12):1540–55. 31709779 10.1002/acr.24042 PMC6884664

[79] Curtis JR, et al. Predicting risk for radiographic damage in rheumatoid arthritis: comparative analysis of the multi-biomarker disease activity score and conventional measures of disease activity in multiple studies. Curr Med Res Opin. 2019;35(9):1483–93. 30777458 10.1080/03007995.2019.1585064

[80] van der Helm-van Mil AH, et al. An evaluation of molecular and clinical remission in rheumatoid arthritis by assessing radiographic progression. Rheumatology (Oxford). 2013;52(5):839–46. 23287359 10.1093/rheumatology/kes378 PMC3630394

[81] Curtis JR, et al. Biomarker-related risk for myocardial infarction and serious infections in patients with rheumatoid arthritis: a population-based study. Ann Rheum Dis. 2018;77(3):386–92. 29269418 10.1136/annrheumdis-2017-211727

[82] Curtis JR, et al. Derivation and internal validation of a multi-biomarker-based cardiovascular disease risk prediction score for rheumatoid arthritis patients. Arthritis Res Ther. 2020;22(1):282. 33276814 10.1186/s13075-020-02355-0 PMC7718706

[83] Lauper K, et al. Effectiveness of TNF-inhibitors, abatacept, IL6-inhibitors and JAK-inhibitors in 31 846 patients with rheumatoid arthritis in 19 registers from the “JAK-pot” collaboration. Ann Rheum Dis. 2022;81(10):1358–66. 35705376 10.1136/annrheumdis-2022-222586 PMC9484385

[84] Mok CC, et al. Safety of the JAK and TNF inhibitors in rheumatoid arthritis: real world data from the Hong Kong Biologics Registry. Rheumatology (Oxford). 2023; May 2:kead198. 10.1093/rheumatology/kead198 37129549

[85] Idriss HT, Naismith JH. TNF alpha and the TNF receptor superfamily: structure-function relationship(s). Microsc Res Tech. 2000;50(3):184–95. 10891884 10.1002/1097-0029(20000801)50:3<184::AID-JEMT2>3.0.CO;2-H

[86] Wang F, et al. Efficacy and safety of tofacitinib, baricitinib, and upadacitinib for rheumatoid arthritis: a systematic review and meta-analysis. Mayo Clin Proc. 2020;95(7):1404–19. 32499126 10.1016/j.mayocp.2020.01.039

[87] Genovese MC, et al. Effect of filgotinib vs placebo on clinical response in patients with moderate to severe rheumatoid arthritis refractory to disease-modifying antirheumatic drug therapy: the FINCH 2 randomized clinical trial. JAMA. 2019;322(4):315–25. 31334793 10.1001/jama.2019.9055 PMC6652745

[88] Charles-Schoeman C, et al. Potential mechanisms leading to the abnormal lipid profile in patients with rheumatoid arthritis versus healthy volunteers and reversal by tofacitinib. Arthritis Rheumatol. 2015;67(3):616–25. 25470338 10.1002/art.38974 PMC5024065

[89] Charles-Schoeman C, et al. Effects of tofacitinib and other DMARDs on lipid profiles in rheumatoid arthritis: implications for the rheumatologist. Semin Arthritis Rheum. 2016;46(1):71–80. 27079757 10.1016/j.semarthrit.2016.03.004

[90] Ytterberg SR, Bhatt DL, Connell CA. Cardiovascular and cancer risk with tofacitinib in rheumatoid arthritis. Reply N Engl J Med. 2022;386(18):1768. 35507493 10.1056/NEJMc2202778

[91] Khosrow-Khavar F, et al. Tofacitinib and risk of cardiovascular outcomes: results from the Safety of TofAcitinib in Routine care patients with Rheumatoid Arthritis (STAR-RA) study. Ann Rheum Dis. 2022;81(6):798–804. 35027405 10.1136/annrheumdis-2021-221915 PMC9117457

[92] Roubille C, et al. The effects of tumour necrosis factor inhibitors, methotrexate, non-steroidal anti-inflammatory drugs and corticosteroids on cardiovascular events in rheumatoid arthritis, psoriasis and psoriatic arthritis: a systematic review and meta-analysis. Ann Rheum Dis. 2015;74(3):480–9. 25561362 10.1136/annrheumdis-2014-206624 PMC4345910

[93] Ljung L, et al. Response to biological treatment and subsequent risk of coronary events in rheumatoid arthritis. Ann Rheum Dis. 2016;75(12):2087–94. 26984007 10.1136/annrheumdis-2015-208995

[94] Dixon WG, et al. Reduction in the incidence of myocardial infarction in patients with rheumatoid arthritis who respond to anti-tumor necrosis factor alpha therapy: results from the British Society for Rheumatology Biologics Register. Arthritis Rheum. 2007;56(9):2905–12. 17763428 10.1002/art.22809 PMC2435427

[95] Aletaha D, et al. 2010 rheumatoid arthritis classification criteria: an American College of Rheumatology/European League Against Rheumatism collaborative initiative. Ann Rheum Dis. 2010;69(9):1580–8. 20699241 10.1136/ard.2010.138461

[96] Ronnelid J, Turesson C, Kastbom A. Autoantibodies in rheumatoid arthritis—laboratory and clinical perspectives. Front Immunol. 2021;12: 685312. 34054878 10.3389/fimmu.2021.685312 PMC8161594

[97] Quisi A, Alici G. The relationship between serum rheumatoid factor level and no-reflow phenomenon in patients with acute ST-segment elevation myocardial infarction undergoing primary percutaneous coronary intervention. J Clin Lab Anal. 2018;32(9): e22598. 29943408 10.1002/jcla.22598 PMC6816820

[98] Tomasson G, et al. Effect of rheumatoid factor on mortality and coronary heart disease. Ann Rheum Dis. 2010;69(9):1649–54. 19628821 10.1136/ard.2009.110536 PMC3653630

[99] Tan EM, Smolen JS. Historical observations contributing insights on etiopathogenesis of rheumatoid arthritis and role of rheumatoid factor. J Exp Med. 2016;213(10):1937–50. 27621417 10.1084/jem.20160792 PMC5030811

[100] Edwards CJ, et al. The autoantibody rheumatoid factor may be an independent risk factor for ischaemic heart disease in men. Heart. 2007;93(10):1263–7. 17550930 10.1136/hrt.2006.097816 PMC2000921

[101] Holmqvist ME, et al. Rapid increase in myocardial infarction risk following diagnosis of rheumatoid arthritis amongst patients diagnosed between 1995 and 2006. J Int Med. 2010;268(6):578–85. 10.1111/j.1365-2796.2010.02260.x 20698926

[102] Kato H, Yamakawa M, Ogino T. Complement mediated vascular endothelial injury in rheumatoid nodules: a histopathological and immunohistochemical study. J Rheumatol. 2000;27(8):1839–47. 10955322

[103] Majka DS, et al. Association of rheumatoid factors with subclinical and clinical atherosclerosis in African American Women: the multiethnic study of atherosclerosis. Arthritis Care Res (Hoboken). 2017;69(2):166–74. 27159164 10.1002/acr.22930

[104] Myasoedova E, et al. The influence of rheumatoid arthritis disease characteristics on heart failure. J Rheumatol. 2011;38(8):1601–6. 21572155 10.3899/jrheum.100979 PMC3337549

[105] Zhu Z, et al. Serum rheumatoid factor levels at acute phase of ischemic stroke are associated with poststroke cognitive impairment. J Stroke Cerebrovasc Dis. 2019;28(4):1133–40. 30661971 10.1016/j.jstrokecerebrovasdis.2018.12.049

[106] Okada Y, et al. Genetics of rheumatoid arthritis contributes to biology and drug discovery. Nature. 2014;506(7488):376–81. 24390342 10.1038/nature12873 PMC3944098

[107] Folkersen L, et al. Mapping of 79 loci for 83 plasma protein biomarkers in cardiovascular disease. PLoS Genet. 2017;13(4): e1006706. 28369058 10.1371/journal.pgen.1006706 PMC5393901

[108] Soderholm M, et al. Genome-wide association meta-analysis of functional outcome after ischemic stroke. Neurology. 2019;92(12):e1271–83. 30796134 10.1212/WNL.0000000000007138 PMC6511098

[109] Jia Y, et al. Associations of rheumatoid factor, rheumatoid arthritis, and interleukin-6 inhibitor with the prognosis of ischemic stroke: a prospective multicenter cohort study and mendelian randomization analysis. Transl Stroke Res. 2023; May 31. 10.1007/s12975-023-01161-5 37256492

[110] Su JH, et al. Interleukin-6: a novel target for cardio-cerebrovascular diseases. Front Pharmacol. 2021;12: 745061. 34504432 10.3389/fphar.2021.745061 PMC8421530

[111] Han S, et al. Radial BMD and serum CTX-I can predict the progression of carotid plaque in rheumatoid arthritis: a 3-year prospective cohort study. Arthritis Res Ther. 2021;23(1):258. 34641970 10.1186/s13075-021-02642-4 PMC8513174

[112] Pulito-Cueto V, et al. Anti-IL-6 therapy reduces leptin serum levels in patients with rheumatoid arthritis. Clin Exp Rheumatol. 2020;38(6):1201–5. 32452351

[113] Nikolopoulos D, et al. Microglia activation in the presence of intact blood-brain barrier and disruption of hippocampal neurogenesis via IL-6 and IL-18 mediate early diffuse neuropsychiatric lupus. Ann Rheum Dis. 2023;82(5):646–57. 36898766 10.1136/ard-2022-223506 PMC10176423

[114] Pawluk H, et al. Effect of IL-6 and hsCRP serum levels on functional prognosis in stroke patients undergoing IV-thrombolysis: retrospective analysis. Clin Interv Aging. 2020;15:1295–303. 32821090 10.2147/CIA.S258381 PMC7418453

[115] Cimmino G, et al. Vitamin D inhibits IL-6 pro-atherothrombotic effects in human endothelial cells: a potential mechanism for protection against COVID-19 infection? J Cardiovasc Dev Dis. 2022;9(1):27. 35050236 10.3390/jcdd9010027 PMC8781542

[116] Autieri MV. IL-19 and other IL-20 family member cytokines in vascular inflammatory diseases. Front Immunol. 2018;9:700. 29681905 10.3389/fimmu.2018.00700 PMC5897441

[117] Hsu YH, et al. Anti-IL-20 monoclonal antibody inhibited inflammation and protected against cartilage destruction in murine models of osteoarthritis. PLoS ONE. 2017;12(4): e0175802. 28426699 10.1371/journal.pone.0175802 PMC5398531

[118] Kwan J, et al. IL-6 is a predictive biomarker for stroke associated infection and future mortality in the elderly after an ischemic stroke. Exp Gerontol. 2013;48(9):960–5. 23872300 10.1016/j.exger.2013.07.003

[119] Shaafi S, et al. Interleukin-6, a reliable prognostic factor for ischemic stroke. Iran J Neurol. 2014;13(2):70–6. 25295149 PMC4187333

[120] Penner LS, et al. Does coprescribing nonsteroidal anti-inflammatory drugs and oral anticoagulants increase the risk of major bleeding, stroke and systemic embolism? Br J Clin Pharmacol. 2022;88(11):4789–811. 35484847 10.1111/bcp.15371 PMC9796910

[121] Bresalier RS, et al. Cardiovascular events associated with rofecoxib in a colorectal adenoma chemoprevention trial. N Engl J Med. 2005;352(11):1092–102. 15713943 10.1056/NEJMoa050493

[122] Baoqi Y, et al. Effect of anti-rheumatic drugs on cardiovascular disease events in rheumatoid arthritis. Front Cardiovasc Med. 2021;8: 812631. 35187113 10.3389/fcvm.2021.812631 PMC8850698

[123] Bindu S, Mazumder S, Bandyopadhyay U. Non-steroidal anti-inflammatory drugs (NSAIDs) and organ damage: a current perspective. Biochem Pharmacol. 2020;180: 114147. 32653589 10.1016/j.bcp.2020.114147 PMC7347500

[124] Curtis E, et al. Safety of cyclooxygenase-2 inhibitors in osteoarthritis: outcomes of a systematic review and meta-analysis. Drugs Aging. 2019;36(Suppl 1):25–44. 31073922 10.1007/s40266-019-00664-x PMC6509094

[125] Moore N. Coronary risks associated with diclofenac and other NSAIDs: an update. Drug Saf. 2020;43(4):301–18. 31916080 10.1007/s40264-019-00900-8

[126] Schink T, et al. Risk of ischemic stroke and the use of individual non-steroidal anti-inflammatory drugs: a multi-country European database study within the SOS Project. PLoS ONE. 2018;13(9): e0203362. 30231067 10.1371/journal.pone.0203362 PMC6145581

[127] Solomon SD, et al. Cardiovascular risk associated with celecoxib in a clinical trial for colorectal adenoma prevention. N Engl J Med. 2005;352(11):1071–80. 15713944 10.1056/NEJMoa050405

[128] Arber N, et al. Celecoxib for the prevention of colorectal adenomatous polyps. N Engl J Med. 2006;355(9):885–95. 16943401 10.1056/NEJMoa061652

[129] Gudbjornsson B, et al. Rofecoxib, but not celecoxib, increases the risk of thromboembolic cardiovascular events in young adults-a nationwide registry-based study. Eur J Clin Pharmacol. 2010;66(6):619–25. 20157701 10.1007/s00228-010-0789-2

[130] Gunter BR, et al. Non-steroidal anti-inflammatory drug-induced cardiovascular adverse events: a meta-analysis. J Clin Pharm Ther. 2017;42(1):27–38. 28019014 10.1111/jcpt.12484

[131] Zhang M, et al. NSAID-associated small intestinal injury: an overview from animal model development to pathogenesis, treatment, and prevention. Front Pharmacol. 2022;13: 818877. 35222032 10.3389/fphar.2022.818877 PMC8864225

[132] Varas-Lorenzo C, et al. Stroke risk and NSAIDs: a systematic review of observational studies. Pharmacoepidemiol Drug Saf. 2011;20(12):1225–36. 21971833 10.1002/pds.2227

[133] Caughey GE, et al. Stroke risk and NSAIDs: an Australian population-based study. Med J Aust. 2011;195(9):525–9. 22060087 10.5694/mja11.10055

[134] Trelle S, et al. Cardiovascular safety of non-steroidal anti-inflammatory drugs: network meta-analysis. BMJ. 2011;342: c7086. 21224324 10.1136/bmj.c7086 PMC3019238

[135] Fanelli A, et al. Cardiovascular and cerebrovascular risk with nonsteroidal anti-inflammatory drugs and cyclooxygenase 2 inhibitors: latest evidence and clinical implications. Ther Adv Drug Saf. 2017;8(6):173–82. 28607667 10.1177/2042098617690485 PMC5455842

[136] Masclee GMC, et al. Risk of acute myocardial infarction during use of individual NSAIDs: a nested case-control study from the SOS project. PLoS ONE. 2018;13(11): e0204746. 30383755 10.1371/journal.pone.0204746 PMC6211656

[137] White WB, et al. Risk of cardiovascular events in patients receiving celecoxib: a meta-analysis of randomized clinical trials. Am J Cardiol. 2007;99(1):91–8. 17196469 10.1016/j.amjcard.2006.07.069

[138] McGettigan P, Henry D. Cardiovascular risk with non-steroidal anti-inflammatory drugs: systematic review of population-based controlled observational studies. PLoS Med. 2011;8(9): e1001098. 21980265 10.1371/journal.pmed.1001098 PMC3181230

[139] Solomon DH, et al. Immunosuppressive medications and hospitalization for cardiovascular events in patients with rheumatoid arthritis. Arthritis Rheum. 2006;54(12):3790–8. 17136752 10.1002/art.22255

[140] Ferreira MB, et al. Sex differences in circulating proteins of patients with rheumatoid arthritis: a cohort study. Int J Rheum Dis. 2022;25(6):669–77. 35429115 10.1111/1756-185X.14323

[141] Park K, Bavry AA. Risk of stroke associated with nonsteroidal anti-inflammatory drugs. Vasc Health Risk Manag. 2014;10:25–32. 24421643 10.2147/VHRM.S54159 PMC3888349

[142] Crofford LJ. Use of NSAIDs in treating patients with arthritis. Arthritis Res Ther. 2013;15(Suppl 3):S2. 24267197 10.1186/ar4174 PMC3891482

[143] Karimi A, et al. A comprehensive insight into effects of resveratrol on molecular mechanism in rheumatoid arthritis: a literature systematic review. Int J Rheum Dis. 2022;25(8):827–43. 35754354 10.1111/1756-185X.14356

[144] Ricciotti E, FitzGerald GA. Prostaglandins and inflammation. Arter Thromb Vasc Biol. 2011;31(5):986–1000. 10.1161/ATVBAHA.110.207449 PMC3081099 21508345

[145] Tsai TF, et al. Recommendations for psoriatic arthritis management: a joint position paper of the Taiwan Rheumatology Association and the Taiwanese Association for Psoriasis and Skin Immunology. J Formos Med Assoc. 2021;120(3):926–38. 33012636 10.1016/j.jfma.2020.08.026

[146] Zheng M, et al. Application of nanomaterials in the treatment of rheumatoid arthritis. RSC Adv. 2021;11(13):7129–37. 35423287 10.1039/d1ra00328c PMC8695100

[147] Andersohn F, et al. Cyclooxygenase-2 selective nonsteroidal anti-inflammatory drugs and the risk of ischemic stroke: a nested case-control study. Stroke. 2006;37(7):1725–30. 16728684 10.1161/01.STR.0000226642.55207.94

[148] Anuncibay-Soto B, Font-Belmonte E, Fernandez-Lopez A. Combining anti-inflammatory and unfolding protein responses to fight stroke. Neural Regen Res. 2019;14(3):450–1. 30539812 10.4103/1673-5374.245468 PMC6334615

[149] Bakhriansyah M, et al. Cyclo-oxygenase selectivity and chemical groups of nonsteroidal anti-inflammatory drugs and the frequency of reporting hypersensitivity reactions: a case/noncase study in VigiBase. Fundam Clin Pharmacol. 2019;33(5):589–600. 30860620 10.1111/fcp.12463 PMC6850345

[150] Jung JY, et al. Body mass index and glucocorticoid dose contribute to subclinical atherosclerosis in Korean patients with systemic lupus erythematosus: a prospective 4 year follow-up study. Int J Rheum Dis. 2019;22(8):1410–8. 31050219 10.1111/1756-185X.13588

[151] Wu F, et al. Inflammation and oxidative stress: potential targets for improving prognosis after subarachnoid hemorrhage. Front Cell Neurosci. 2021;15: 739506. 34630043 10.3389/fncel.2021.739506 PMC8497759

[152] Gong L, et al. Celecoxib pathways: pharmacokinetics and pharmacodynamics. Pharmacogenet Genom. 2012;22(4):310–8. 10.1097/FPC.0b013e32834f94cb PMC3303994 22336956

[153] Chen AI, et al. Celecoxib and Etoricoxib may reduce risk of ischemic stroke in patients with rheumatoid arthritis: a nationwide retrospective cohort study. Front Neurol. 2022;13:1018521. 36341096 10.3389/fneur.2022.1018521 PMC9630581

[154] Danda D, et al. How safe is Celecoxib for Asian-Indian patients with rheumatic diseases? Int J Rheum Dis. 2013;16(1):24–9. 23441769 10.1111/1756-185X.12043

[155] Lin YW, Wang JY, Lin MH. Stroke risk associated with NSAIDs uses in women with dysmenorrhea: a population-based cohort study. PLoS ONE. 2021;16(11): e0259047. 34767568 10.1371/journal.pone.0259047 PMC8589167

[156] Turner RJ, et al. A substance P antagonist improves outcome when administered 4 h after onset of ischaemic stroke. Brain Res. 2011;1393:84–90. 21466790 10.1016/j.brainres.2011.03.066

[157] Bruno G, et al. The role of substance P in cerebral ischemia. Int J Immunopathol Pharmacol. 2003;16(1):67–72. 12578734 10.1177/039463200301600110

[158] O’Connor TM, et al. The role of substance P in inflammatory disease. J Cell Physiol. 2004;201(2):167–80. 15334652 10.1002/jcp.20061

[159] Janelsins BM, et al. Neurokinin-1 receptor agonists bias therapeutic dendritic cells to induce type 1 immunity by licensing host dendritic cells to produce IL-12. Blood. 2013;121(15):2923–33. 23365459 10.1182/blood-2012-07-446054 PMC3624938

[160] Goto T, Tanaka T. Tachykinins and tachykinin receptors in bone. Microsc Res Tech. 2002;58(2):91–7. 12203708 10.1002/jemt.10123

[161] Munoz M, Covenas R. Involvement of substance P and the NK-1 receptor in cancer progression. Peptides. 2013;48:1–9. 23933301 10.1016/j.peptides.2013.07.024

[162] Munoz M, Covenas R. Involvement of substance P and the NK-1 receptor in human pathology. Amino Acids. 2014;46(7):1727–50. 24705689 10.1007/s00726-014-1736-9

[163] Liu X, et al. Antagonism of NK-1R using aprepitant suppresses inflammatory response in rheumatoid arthritis fibroblast-like synoviocytes. Artif Cells Nanomed Biotechnol. 2019;47(1):1628–34. 31010320 10.1080/21691401.2019.1573177

[164] Barbosa-Cobos RE, et al. Serum substance P: an indicator of disease activity and subclinical inflammation in rheumatoid arthritis. Clin Rheumatol. 2018;37(4):901–8. 29256110 10.1007/s10067-017-3929-6

[165] Hong HS, Son Y. Substance P ameliorates collagen II-induced arthritis in mice via suppression of the inflammatory response. Biochem Biophys Res Commun. 2014;453(1):179–84. 25264193 10.1016/j.bbrc.2014.09.090

[166] Jiang MH, et al. Substance P induces M2-type macrophages after spinal cord injury. NeuroReport. 2012;23(13):786–92. 22825006 10.1097/WNR.0b013e3283572206

[167] Origuchi T, et al. Reduction in serum levels of substance P in patients with rheumatoid arthritis by etanercept, a tumor necrosis factor inhibitor. Mod Rheumatol. 2011;21(3):244–50. 21188454 10.1007/s10165-010-0384-5

[168] Bland JH, Eddy WM. Hemiplegia and rheumatoid hemiarthritis. Arthritis Rheum. 1968;11(1):72–80. 5643259 10.1002/art.1780110110

[169] Newman M. The process of recovery after hemiplegia. Stroke. 1972;3(6):702–10. 4639102 10.1161/01.str.3.6.702

[170] Marshall KW, Chiu B, Inman RD. Substance P and arthritis: analysis of plasma and synovial fluid levels. Arthritis Rheum. 1990;33(1):87–90. 1689161 10.1002/art.1780330111

[171] Furman D, et al. Chronic inflammation in the etiology of disease across the life span. Nat Med. 2019;25(12):1822–32. 31806905 10.1038/s41591-019-0675-0 PMC7147972

[172] Putaala J, et al. Searching for explanations for cryptogenic stroke in the young: revealing the triggers, causes, and outcome (SECRETO): rationale and design. Eur Stroke J. 2017;2(2):116–25. 31008307 10.1177/2396987317703210 PMC6453214

[173] Jin R, et al. Role of inflammation and its mediators in acute ischemic stroke. J Cardiovasc Transl Res. 2013;6(5):834–51. 24006091 10.1007/s12265-013-9508-6 PMC3829610

[174] Fatkhullina AR, Peshkova IO, Koltsova EK. The role of cytokines in the development of atherosclerosis. Biochemistry (Mosc). 2016;81(11):1358–70. 27914461 10.1134/S0006297916110134 PMC5471837

[175] Lip GYH, et al. Integrated care for optimizing the management of stroke and associated heart disease: a position paper of the European Society of Cardiology Council on Stroke. Eur Heart J. 2022;43(26):2442–60. 35552401 10.1093/eurheartj/ehac245 PMC9259378

[176] Bacani AK, et al. Noncardiac vascular disease in rheumatoid arthritis: increase in venous thromboembolic events? Arthritis Rheum. 2012;64(1):53–61. 21905005 10.1002/art.33322 PMC3474372

[177] Turesson C, Jarenros A, Jacobsson L. Increased incidence of cardiovascular disease in patients with rheumatoid arthritis: results from a community based study. Ann Rheum Dis. 2004;63(8):952–5. 15051620 10.1136/ard.2003.018101 PMC1755101

[178] Westerlind H, et al. The association between autoantibodies and risk for venous thromboembolic events among patients with rheumatoid arthritis. Rheumatology (Oxford). 2023;62(6):2106–12. 36255271 10.1093/rheumatology/keac601 PMC10234203

[179] Mantel A, et al. Risk factors for the rapid increase in risk of acute coronary events in patients with new-onset rheumatoid arthritis: a nested case-control study. Arthritis Rheumatol. 2015;67(11):2845–54. 26138387 10.1002/art.39267

[180] Westerlind H, et al. Anti-citrullinated protein antibody specificities, rheumatoid factor isotypes, and incident cardiovascular events in patients with rheumatoid arthritis. Arthritis Rheumatol. 2020;72(10):1658–67. 32475073 10.1002/art.41381

[181] Meznerics FA, et al. Multibiomarker disease activity score: an objective tool for monitoring rheumatoid arthritis? A systematic review and meta-analysis. Rheumatology (Oxford). 2023;62(6):2048–59. 36575983 10.1093/rheumatology/keac715 PMC10234206

[182] Johnson TM, et al. Correlation of the multi-biomarker disease activity score with rheumatoid arthritis disease activity measures: a systematic review and meta-analysis. Arthritis Care Res (Hoboken). 2019;71(11):1459–72. 30320973 10.1002/acr.23785 PMC6465168

[183] Ortega-Hernandez OD, et al. Cardiovascular disease is associated with extra-articular manifestations in patients with rheumatoid arthritis. Clin Rheumatol. 2009;28(7):767–75. 19277815 10.1007/s10067-009-1145-8

[184] Arida A, et al. Systemic inflammatory response and atherosclerosis: the paradigm of chronic inflammatory rheumatic diseases. Int J Mol Sci. 2018;19(7):1890. 29954107 10.3390/ijms19071890 PMC6073407

[185] Giachi A, Cugno M, Gualtierotti R. Disease-modifying anti-rheumatic drugs improve the cardiovascular profile in patients with rheumatoid arthritis. Front Cardiovasc Med. 2022;9:1012661. 36352850 10.3389/fcvm.2022.1012661 PMC9637771

